# Incidence of PJI in Total Knee Arthroplasty Patients Following Expanded Gram-Negative Antibiotic Prophylactic Protocol

**DOI:** 10.3390/microorganisms13051002

**Published:** 2025-04-27

**Authors:** Anzar Sarfraz, Cameron Bussey-Sutton, Emily M. Ronan, Farouk Khury, Joseph A. Bosco, Ran Schwarzkopf, Vinay K. Aggarwal

**Affiliations:** 1 Division of Adult Reconstruction Hip/Knee Replacement, Department of Orthopedic Surgery, NYU Grossman School of Medicine, NYU Langone Health, Adult Reconstruction Bellevue Hospital, 462 First Ave (BHC), CD Bldg Room 4-85, New York, NY 10016, USA; sarfrazanzar80@gmail.com (A.S.); fkhury@gmail.com (F.K.); schwarzk@gamil.com (R.S.); 2 Division of Orthopedic Surgery, Rambam Medical Center, The Ruth and Bruce Rappaport Faculty of Medicine, Haifa 3109601, Israel

**Keywords:** knee, gram-negative bacteria, prophylaxis, prosthesis, joint infections, implants

## Abstract

The efficacy of “Expanded Gram-Negative Antimicrobial Prophylaxis” (EGNAP) in preventing postoperative infections has been previously reported in total hip arthroplasty (THA). However, it remains unclear as to whether these benefits extend to total knee arthroplasty (TKA). This study investigated whether adding EGNAP to our institution’s preoperative antibiotic prophylaxis protocol would affect periprosthetic joint infection (PJI) risk in TKA patients. We retrospectively reviewed 10,666 elective, unilateral, primary TKA cases performed at a single-specialty tertiary academic hospital from 2018 to 2022. Before June 2021, all patients received 2 g of cefazolin for 24 h as part of the prophylactic antibiotic protocol. After June 2021, gentamicin or aztreonam (EGNAP) was added to the protocol for all TKA patients. Patients were grouped based on whether they received EGNAP or not (control group) before surgery. The groups were propensity score-matched in a 2:1 ratio. PJI and nephrotoxicity (using RIFLE criteria) risk was compared. After matching, the final study population consisted of 3007 patients in the non-EGNAP group and 1503 patients in the EGNAP group. There was no significant difference between the EGNAP and no EGNAP groups in the overall incidence of PJI (1.9% vs. 2.0%; *p* = 0.111) or the incidence of Gram-positive PJIs (0.3% vs. 0.8%; *p* = 0.103). The incidence of Gram-negative PJIs was 0.5% in the EGNAP group and 0.4% in the no EGNAP group, which was also not different between the groups (*p* = 0.692). There were no differences in nephrotoxicity between groups (*p* = 0.521). The addition of EGNAP to the antibiotic prophylactic protocol prior to TKA had no effect on overall or Gram-negative PJI risk in TKA patients. The findings of this study suggest that while EGNAP is safe to use and has minimal nephrotoxic effects, its prophylactic benefits do not extend to the primary TKA population. This may be attributed to the generally low rate of Gram-negative infections in TKA patients, where adding EGNAP does not provide a clear advantage in reducing the risk of such infections, unlike its potential benefits in primary THA population. This study investigates the effects of using prophylactic Gram-negative antibiotics prior to TKA and shows that though it is safe to use, Gram-negative bacterial coverage may have no impact on postoperative infection incidence.

## 1. Introduction

Preventing infection is a top priority after total knee arthroplasty (TKA). However, periprosthetic joint infection (PJI) still occurs in 1% to 4% of cases [1,2,3,4]. Although several factors influence PJI prevention, perioperative antibiotic prophylaxis is critical and remains the cornerstone of infection control [5].

First-generation cephalosporins have long been the recommended prophylactic antibiotics for TKA and total hip arthroplasty (THA) [6,7,8,9,10,11]. Cefazolin in particular has a broad coverage and has good tissue penetration into bone, synovium, muscle, and hematoma [12,13]. When administered within sixty minutes of incision or tourniquet inflation, cephalosporins have been reported to have an effective coverage against Gram-positive organisms, which remain the most common cause of deep surgical site infection (SSI) and PJI [14,15,16]. However, the evolving landscape of pathogens presents a growing challenge. Gram-negative bacteria (GNB) have become more dominant and virulent in PJIs in recent years [17,18,19]. Additionally, a growing resistance to cefazolin, particularly in Gram-negative species, has been observed nationwide, highlighting a need to reassess prophylactic strategies [2,20]. This is especially concerning in TKA, where the prevalence of Gram-negative infections is low, and delayed recognition or inadequate prophylaxis may lead to disproportionately sever outcomes.

Current guidelines from the American Society of Health-System Pharmacists emphasize the importance of tailoring antibiotic prophylaxis to local resistance patterns and specific institutional needs [11]. In a recent systematic review of 593 patients with PJIs, González et al. found that 29% (169 of 593) were knee infections caused by GNB [21]. Given the growing impact of Gram-negative bacteria in PJIs, broader antimicrobial coverage is becoming increasingly important. In a retrospective study, Bosco et al. recommended adding weight-based high-dose gentamicin (or aztreonam for patients with contraindications to gentamicin) to the prophylactic antibiotic protocol before THA. This “Expanded Gram-Negative Antimicrobial Prophylaxis” (EGNAP) approach significantly reduced the overall infection rate (from 1.19% to 0.55%) and Gram-negative infection rate (from 0.32% to 0%) in their THA cohort [22]. Despite the promising data from THA, the application of EGNAP in TKA remains underexplored. The anatomic, biomechanical, and procedural differences between hip and knee arthroplasty warrant independent evaluation of this approach in the TKA setting.

With these considerations in mind, we asked two questions: First, does incorporating EGNAP into the preoperative antibiotic protocol reduce the incidence of PJI in TKA patients, similar to the benefits observed in primary THA? Second, does adding EGNAP pose risks, such as acute kidney injury, that might offset its potential benefits in preventing PJI in TKA?

## 2. Materials and Methods

### 2.1. Study Design

After obtaining institutional review board (IRB) approval, we retrospectively reviewed a registry of 10,666 elective, unilateral, primary TKA cases performed at a single-specialty urban academic hospital from January 2018 to March 2022. Patients were included if their surgical indication was degenerative joint disease (DJD) from osteoarthritis, inflammatory arthritis, post-traumatic arthritis, or other causes of DJD of the affected knee. Inclusion criteria also require patients to have a minimum of 1 year of follow-up. Patients were excluded if they underwent a non-elective or simultaneously performed bilateral procedure on the same day.

Preoperatively, patients who tested positive for Staphylococcus aureus underwent preoperative nasal decolonization with chlorhexidine wipes applied from chin to toes on the morning of surgery and povidone–iodine ointment to the nares 1–6 h preoperatively. All primary TKAs were conducted in standard operating rooms with consistent staffing and personnel across all investigated cases. All scrubbed personnel were mandated to wear a surgical helmet and a body exhaust suit. Before June 2021, all patients received either 2 g of cefazolin for 24 h, a single preoperative dose of 15–20 mg/kg of vancomycin if MRSA-positive, or a single preoperative dose of 900 mg of clindamycin for those with severe penicillin or cephalosporin allergies. After June 2021, our protocol was adjusted by adding Gram-negative coverage with 3–5 mg/kg of gentamicin or 2 g of aztreonam for patients aged 75 years and older, weighing ≥120 kg, or with creatinine clearance <20 mL/min. All 10,666 patients received this prophylactic protocol, and the only difference in the non-EGNAP (control group) and the EGNAP group was the addition of Gram-negative coverage within 12 h before surgery.

Intraoperatively, the VIP protocol was followed in the cases of high-risk TKA patients (BMI > 40 kg/m^2^, active smoker, ASA ≥ 3, immunosuppression, MRSA colonization, revision surgery for previous arthroplasty), as defined by Iorio et al. [23]. The protocol includes lavage with a 0.35% povidone–iodine solution (17.5 mL in 500 mL saline), which remains in place for 3 min after final implantation [24]. Following this, pulsed irrigation is performed using 1 L of sterile saline. During wound closure, 1 g of vancomycin powder is applied deep to the fascia, with an additional 1 g placed superficially [25]. The decision to rub the antibiotic into the muscle, fascia, and subcutaneous tissue was left to the surgeon’s discretion and was not documented. Lastly, wound closure techniques and dressing protocols remained unchanged throughout the study period. With regards to DVT prophylaxis, starting May 2014, the institution recommends the use of aspirin as a prophylactic agent in TJA.

### 2.2. Data Collection

Baseline characteristics were collected for each patient, including gender, age at surgery, smoking status, race, American Society of Anesthesiologists (ASA) score, body mass index (BMI), Charlson Comorbidity Index (CCI) score, and diabetic status. Infection information, such as diagnosis of PJI, laboratory markers, synovial fluid analysis, culture positivity, causative organism type, and intraoperative findings, were also collected. At our institution, the diagnosis of PJI is made by an arthroplasty trained surgeon on a case-by-case basis, following established clinical criteria (ICM-2018) [26]. Laboratory results including pre- and postoperative creatinine and estimated glomerular filtration rates (EGFR) were also collected. The mean follow-up time was 2.9 years (SD 1.1) for the non-EGNAP group and 2.4 years (SD 1.0) for the EGNAP group.

### 2.3. Nephrotoxicity Calculation

To calculate the change in creatinine levels (mg/dL) per patient pre- and postoperatively, we took the average mean of all creatinine levels within one year preoperatively and took the highest creatinine level that resulted within one week postoperatively. The change in creatinine was reported as the ratio of the postoperative creatinine value to the preoperative creatinine value; this ratio was calculated by dividing postoperative value by the preoperative value. Risk injury failure loss and end stage kidney disease (RIFLE) criteria were used to classify patients as either at risk, injury, or failure. Patients were classified as at risk if their creatinine ratio was >1.5, at injury if their creatinine ratio was >2, and at failure if their creatinine ratio was >3. To calculate the change in EGFR levels (mL/min) per patient pre- and postoperatively, we took the average mean of all EGFR results within one year preoperatively and took the lowest EGFR result within one week postoperatively. The ratio was calculated by dividing this postoperative value by the preoperative value. Once again, patients were classified by RIFLE criteria. Patients were at risk if their EGFR ratio was <0.75, at injury if their EGFR ratio was <0.5, and at failure if their EGFR ratio was <0.25. Lastly, having two RIFLE classifications per patient (one via creatinine labs and one via EGFR labs), we picked the most critical result as the overall RIFLE classification for the patient. For example, if the patient’s EGFR ratio was 0.70 (RIFLE classification of risk) but their creatinine ratio was 2.1 (RIFLE classification of injury), the patient was classified as at injury.

### 2.4. Data Analysis

All data analyses were performed using SPSS v25 (IBM Corporation, Armonk, NY, USA). To compare the non-EGNAP and EGNAP groups, propensity score matching was performed based on gender, surgery age, smoking status, ASA, and BMI. After matching, the final study population consisted of 3007 patients in the non-EGNAP group and 1503 patients in the EGNAP group. All continuous variables were presented as means with standard deviations, and categorical variables were presented as frequencies with percentages.

The sample size and power calculation for this study were determined using a two-proportion power analysis, aimed at detecting differences in PJI risks between the non-EGNAP and EGNAP groups. A significance level (α) of 0.05 and a power (1 − β) of 80% were selected to minimize Type I and Type II errors. Using the standard formula for comparing two independent proportions, the required sample size was calculated to be 4510 patients (3007 non-EGNAP and 1503 EGNAP). This sample size was sufficient to ensure adequate statistical power for detecting a meaningful difference in infection incidence between the groups.

The normality of the data distribution was assessed using Levene’s test for equality of variances. A *p*-value greater than 0.05 for all continuous variables indicated that the data followed a normal distribution. To compare demographic variables, we used two-sided independent samples *t*-test to analyze surgery age and BMI and chi square analyses to analyze gender, smoking status, race, ASA score, CCI score, and diabetic status. These analyses were performed both prior to and after propensity score matching. All other variables in this study, such as incidence of PJI, culture positivity, Gram stain, and RIFLE criteria, were analyzed using chi square analyses. A *p*-value of less than 0.05 was considered statistically significant for all analyses in this study.

## 3. Results

Following propensity matching for gender, age, smoking status, ASA, and BMI, this study included a total of 4510 cases. Table 1 represents the baseline characteristics of the non-EGNAP and EGNAP groups before and after propensity matching. The non-EGNAP and EGNAP groups comprised 3007 and 1503 cases, respectively. There were no differences in baseline characteristics between the two groups. The overall occurrence of PJI was not significantly different between the two groups (*p* = 0.101), with PJIs occurring in 37 of 3007 cases (1.2%) in the non-EGNAP group and 30 of 1503 cases (2.0%) in the EGNAP group (Table 2).

The prevalence of PJIs caused by Gram-negative bacteria in the non-EGNAP group was 4 out of 3007 cases (0.1%), which did not significantly differ from the 3 out of 1503 cases (0.2%) in the EGNAP group (*p* = 0.692). Similarly, no significant difference was observed in the occurrence of PJIs caused by Gram-positive bacteria (GPB) between the non-EGNAP group (23 of 3007 cases, 0.8%) and the EGNAP group (19 of 1503 cases, 1.3%) (*p* = 0.103) (Table 2).

A subgroup analysis was performed based on obesity and age. Patients were classified according to WHO obesity classification [27] and age above or below 65 years to identify any patients that could additionally be at high risk. There were no differences in the incidence of PJI between these subgroups (Table 3).

Among the PJIs, Gram-positive organisms, specifically staphylococcus species, were the most predominant organisms identified in both the non-EGNAP group (20 of 37 cases, 54.0%) and the EGNAP group (16 of 30 cases, 53.3%). Gram-negative organisms accounted for 4 of 37 cases (10.8%) in the non-EGNAP group and 3 of 30 cases (10%) in the EGNAP group. Culture-negative PJIs were observed in 11 of 37 cases (29.7%) in the non-EGNAP group and 8 of 30 cases (33.3%) in the EGNAP group. Finally, one PJI in the EGNAP group was due to a fungal infection (Table 4).

Among the PJIs, Gram-positive organisms, specifically staphylococcus species, were the most predominant organisms identified in both the non-EGNAP group (20 of 37 cases, 54.0%) and the EGNAP group (16 of 28 cases, 57.1%). Gram-negative organisms accounted for 4 of 37 cases (10.8%) in the non-EGNAP group and 3 of 28 cases (10.7%) in the EGNAP group. Culture-negative PJIs were observed in 11 of 37 cases (29.7%) in the non-EGNAP group and 8 of 28 cases (28.6%) in the EGNAP group. Finally, one PJI in the EGNAP group was due to a fungal infection (Table 4).

The overall rates of nephrotoxicity were 1.7% (51 of 3007 cases) in the non-EGNAP group and 1.0% (15 of 1503 cases) in the EGNAP group, with no differences observed between the two groups (*p* = 0.521). Furthermore, no difference was observed between the groups across the different severity levels indicated by the RIFLE criteria (Table 5).

## 4. Discussion

In our study, the overall incidence of PJI after TKA was 1.0%, with no differences between patients who did not receive EGNAP compared to those who did. Gram-positive bacteria were the most common causative organism of PJI in both groups (62.2% in the control group vs. 63.3% in the EGNAP group), with staphylococcal species being the most prevalent. These findings were consistent with those of the existing literature [2,3,4]. The incidence of GNB PJI was similarly low in both groups, aligning with the incidence reported by Zmistowski et al., although their study also included THAs [19]. While GNB infections are less frequent, they remain a serious concern due to treatment failure rates of 20% to 30% [19,28]. Unlike Gram-positive PJIs, there is no clear consensus on the best prevention or treatment strategies for GNB infections, underscoring the need for improved protocols [18,28,29]. We designed this study to assess whether adding gentamicin to perioperative antibiotic prophylaxis would reduce the occurrence of GNB PJI in patients undergoing TKA. Our findings indicated that adding gentamicin to antibiotic prophylaxis did not lower the incidence of GNB PJI.

To date, no prospective trials have demonstrated clear superiority of any other antibiotic class over cephalosporins in preventing PJI after TKA [30]. A previous retrospective study reported that changing antimicrobial prophylaxis from cefuroxime to teicoplanin with the addition of gentamicin led to a significant reduction in PJI rates from 2.24% to 0.57% at two years follow-up [31]. However, it is important to highlight that almost half (44%) of this study’s cohort were patients who had undergone hip arthroplasty [31].

Our institution previously observed a similar reduction in postoperative infections following THA when adding weight-based high-dose gentamicin (or aztreonam for patients with contraindications to gentamicin) as part of the perioperative antibiotic prophylactic protocol. Bosco et al. reported a decrease in infection caused by both Gram-positive and Gram-negative organisms with gentamicin use [22]. However, our results suggest that the benefits noted by Azamgarhi and Bosco et al. in the THA population may not extend to an isolated TKA population [22,31]. This could be due to the overall lower incidence of Gram-negative infections in TKA compared to THA [22]. In our study, the overall rate of GNB PJI was 0.1%, with no differences between the control and EGNAP groups. By contrast, Bosco et al. reported a 0.32% GNB infection rate in THA [22]. This difference may be due to the anatomical location of the knee joint relative to the hip. Aboltins et al. reported that Gram-negative infections are more common in the hip than in the knee joint [32]. It is well-established that humans have location-specific microbial colonization, which is influenced by factors such as skin folds and proximity to the genitourinary and gastrointestinal tracts [33]. Contrary to the hip joint, the knee joint is located farther from areas typically colonized by Gram-negative bacteria, such as the gastrointestinal and urinary tracts, thereby reducing the risk of intraoperative contamination [32,34,35]. This location-specific microbial colonization can result in different infection risks, with the knee being less prone to infections caused by Gram-negative bacteria compared to the hip.

Due to the potential nephrotoxic and ototoxic effect of gentamicin [36,37], we monitored serum creatinine and also EGFR using the RIFLE criteria [38] and found no difference in nephrotoxicity rates between patients who received gentamicin and those who did not (Table 5). Azamgarhi et al. likewise found no additional risk of stage 2 or stage 3 acute kidney injury (AKI) in patients receiving gentamicin prophylaxis. They observed some changes in stage 1 AKI, which was linked to the combined nephrotoxic effects of teicoplanin in addition to gentamicin [31]. Although we did not perform formal auditory or vestibular testing, no patients reported hearing or balance problems within the follow-up period, aligning with earlier studies showing that ototoxicity from a single aminoglycoside dose is rare [39]. Moreover, our findings suggest that it is safe to use, with no notable increase in nephrotoxicity or ototoxicity.

## 5. Limitations

This study has several limitations. First, the retrospective design of this study makes it difficult to establish a direct causal relationship between EGNAP and infection rates. However, factors such as surgical site preparation, intraoperative and postoperative wound management, aseptic technique, temperature management, and glycemic control remained consistent across both groups. Second, the relatively low incidence of PJI in both groups limited our power to detect significant differences in infection subtypes, particularly those caused by Gram-negative organisms. This low incidence is likely influenced by the stringent infection control measures at our single-specialty urban academic center, combined with the high-volume experience of our fellowship-trained arthroplasty surgeons. While this strengthens internal validity, it may limit the generalizability of our findings to other institutions with different microbiological profiles or patient populations. Third is the temporal design of the presented data. The EGNAP protocol was introduced for TKAs performed after June 2021, while the non-EGNAP group includes surgeries performed prior to that time. Although this introduces potential biases due to differences in follow-up duration, we took steps to minimize this impact. The follow-up duration for the EGNAP group was updated to February 2025, with a mean follow up of 2.4 years, which was comparable to the 2.9-year follow-up duration of the non-EGNAP group. Fourth, variability in surgical technique, particularly the intraoperative application of antibiotics across different tissue layers, was left to the discretion of the surgeon and not systematically documented. This might introduce potential variability in local antibiotic concentrations. Finally, while a 2:1 propensity score matching ratio may have introduced some potential bias, it was chosen to increase statistical power. This matching ratio allowed us to maintain an adequate sample size and statistical power to detect differences in infection incidence between the EGNAP and non-EGNAP groups.

## 6. Conclusions

In conclusion, while EGNAP has proven to be an effective strategy for reducing infection rates in our THA population, our study found no added benefit of preoperative gentamicin or aztreonam antibiotics in reducing PJI risk in TKA patients. The low incidence of Gram-negative infections in TKA suggests that additional Gram-negative prophylaxis may not provide a clear benefit for most patients. Continued usage of cefazolin and multimodal infection prophylaxis strategies could prove sufficient in the prevention of overall and GNB PJI in TKA.

## Figures and Tables

**Table 1:** Baseline characteristics before and after propensity matching.

	Pre-Propensity Match			Post-Propensity Match		
	Non-EGNAP (n = 8755)	EGNAP (n = 1911)	*p*-Value	Non-EGNAP (n = 3007)	EGNAP (n = 1503)	*p*-Value
Surgery Age, mean ± SD	66.40 ± 9.4	67.41 ± 9.3	<0.001	67.58 ± 9.2	67.60 ± 9.2	0.937
Gender, n (%)	--	--	<0.001	--	--	0.489
Female	5931 (67.7)	1366 (71.5)		901 (30.0)	1069 (71.1)	
Male	2824 (32.3)	545 (28.5)		2106 (70.0)	435 (28.9)	
BMI, mean ± SD	32.39 ± 6.3	32.47 ± 6.4	0.605	32.46 ± 6.1	32.45 ± 6.3	0.982
Smoking status, n (%)	--	--	0.212	--	--	0.855
Never smoker	5031 (57.5)	1099 (57.5)		1779 (59.2)	881 (58.6)	
Former smoker	3221 (36.8)	721 (37.7)		1082 (36.0)	553 (36.8)	
Current smoker	503 (5.7)	91 (4.8)		146 (4.9)	70 (4.7)	
ASA score, n (%)	--	--	0.105	--	--	0.768
1	158 (1.8)	29 (1.5)		50 (1.7)	26 (1.7)	
2	4674 (53.4)	969 (50.7)		1511 (50.2)	755 (50.2)	
3	3764 (43.0)	879 (46.0)		1389 (46.2)	701 (46.6)	
4	159 (1.8)	34 (1.8)		57 (1.9)	22 (1.5)	
Median CCI	3	3	<0.001	3	2	0.313
Diabetic status, n (%)	--	--	<0.001	--	--	0.338
Diabetic	1897 (21.7)	457 (23.9)		2291 (76.2)	1126 (74.9)	
Non-diabetic	6858 (78.3)	1454 (76.1)		716 (23.8)	378 (25.1)	

EGNAP, Gram-negative bacterial coverage; SD, standard deviation; BMI, body mass index; ASA, American Society of Anesthesiologists; CCI, Charlson Comorbidity Index.

**Table 2:** PJI by Gram stain.

	Non-EGNAP (n = 3007)	EGNAP (n = 1503)	*p*-Value
Any PJI, n (%)	37 (1.2)	30 (2.0)	0.101
Culture-negative PJI	11 (0.4)	10 (0.6)	0.467
Culture-positive PJI, n (%)	26 (0.9)	20 (1.3)	0.157
Gram-negative PJI, n (%)	4 (0.1)	3 (0.2)	0.692
Gram-positive PJI, n (%)	23 (0.8)	19 (0.3)	0.103
Fungal PJI, n (%)	0	1 (0.1)	0.333

EGNAP, Gram-negative bacterial coverage; PJI, periprosthetic joint infection.

**Table 3:** Sub-analysis of high risk group.

	Non-EGNAP (n = 3007)	EGNAP (n = 1503)	*p*-Value
Total PJI, n (%)	37	30	--
Age, n (%)			--
>65	20 (54)	19 (63.3)	0.288
<65	17 (46)	11 (36.7)	0.338
BMI, n (%)			--
BMI < 30	8 (21.6)	7 (23.3)	0.397
BMI 30–34.99	14 (37.8)	9 (30)	0.769
BMI 35–39.99	11 (29.7)	9 (30)	0.338
BMI > 40	4 (10.8)	5 (16.6)	0.284

EGNAP, Gram-negative bacterial coverage; PJI, periprosthetic joint infection; BMI, body mass index kg/m^2^.

**Table 4:** PJI rates by organism.

	Non-EGNAP (n = 3007)	EGNAP (n = 1503)	*p*-Value
Total PJI, n	37	30	--
Gram-negative PJI, n (%) *	4 (10.8)	3 (10)	>0.999
Pseudomonas aeruginosa	1 (2.7)	1 (3.3)	--
Escherichia coli	1 (2.7)	0	--
Enterobacter cloacae	1 (2.7)	0	--
Other GNB	1 (2.7)	3 (10)	--
Gram-positive PJI, n (%) **	23 (62.2)	19 (63.3)	0.794
Staphylococcus species	20 (54.0)	16 (53.3)	--
Streptococcus species	2 (5.4)	5 (16.6)	--
Enterococcus faecalis	2 (5.4)	2 (6.6)	--
Other GPB	5 (13.5)	3 (10)	--
Fungal PJI, n (%) ***	0	1 (3.3)	0.431
Candida species	0	1 (3.3)	--
Culture-negative PJI, n (%)	11 (29.7)	10 (33.3)	>0.999

EGNAP, Gram-negative bacterial coverage; PJI, periprosthetic joint infection. * This included three patients who had a polybacterial GNB + GPB. ** This included seven patients who had a polybacterial GPB, three with a polybacterial GNB + GPB, and one with a polybacterial GPB + fungus. *** This included one patient with a polybacterial GPB + fungus.

**Table 5:** Nephrotoxicity.

	Non-EGNAP (n = 3007)	EGNAP (n = 1503)	*p*-Value
Severity of nephrotoxicity by RIFLE criteria, n (%)	51 (1.7%)	15 (1.0%)	0.521
Risk	48 (1.6)	13 (0.9)	0.055
Injury	2 (7 × 10^−4^)	2 (0.1)	0.605
Failure	1 (7 × 10^−4^)	0	0.999

EGNAP, Gram-negative bacterial coverage; RIFLE, risk, injury, failure, loss of kidney function, end-stage kidney disease.

## Data Availability

The original contributions presented in this study are included in the article. Further inquiries can be directed to the corresponding author.
